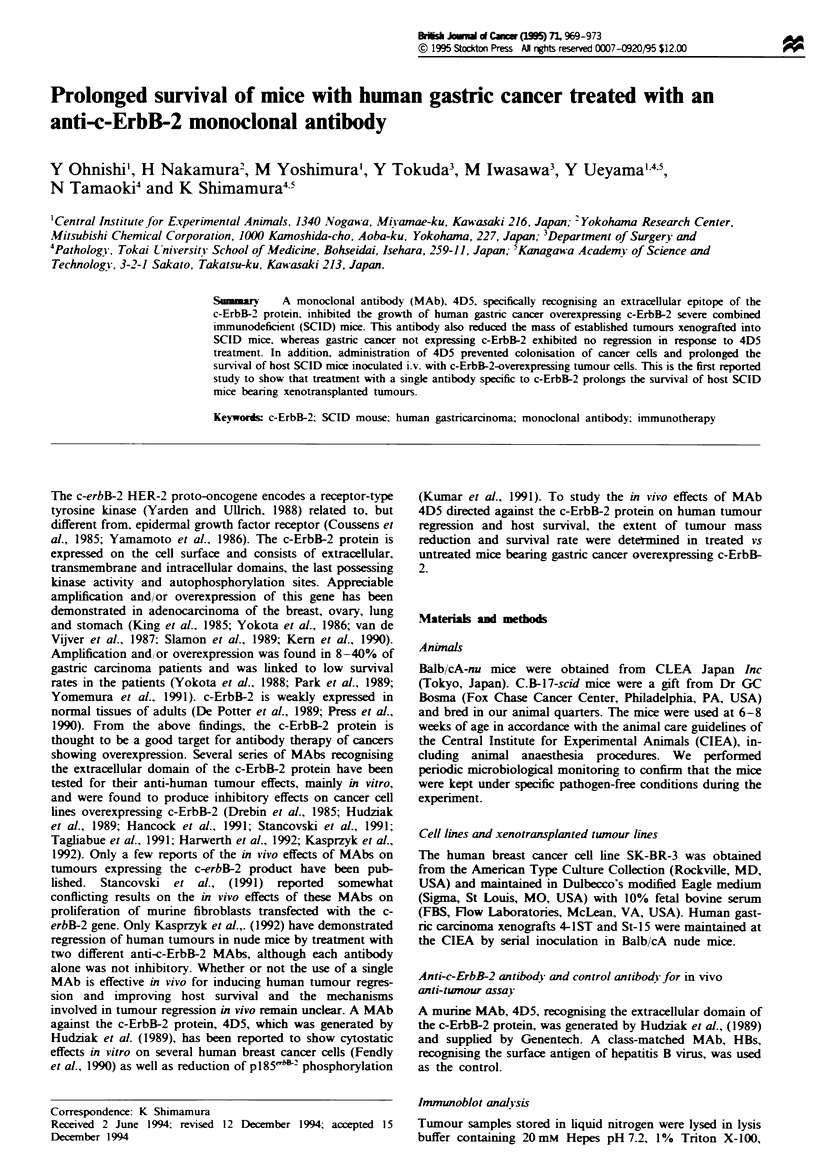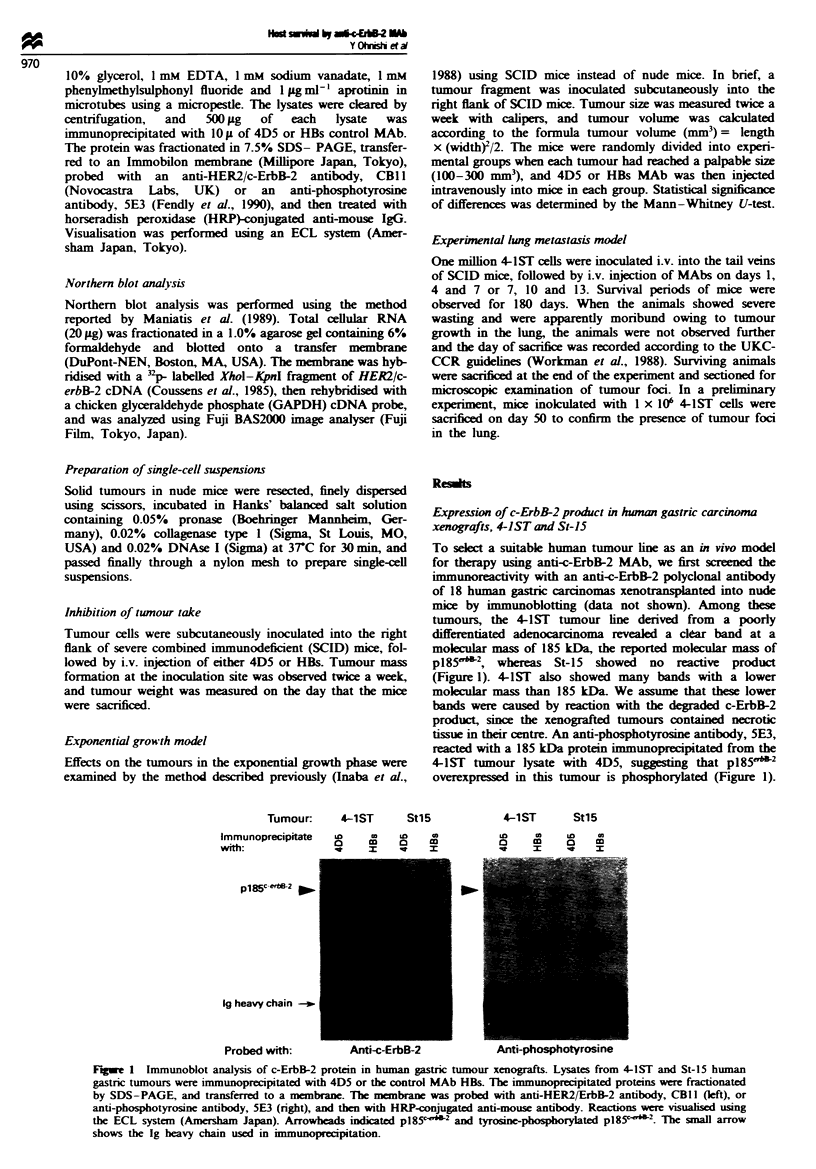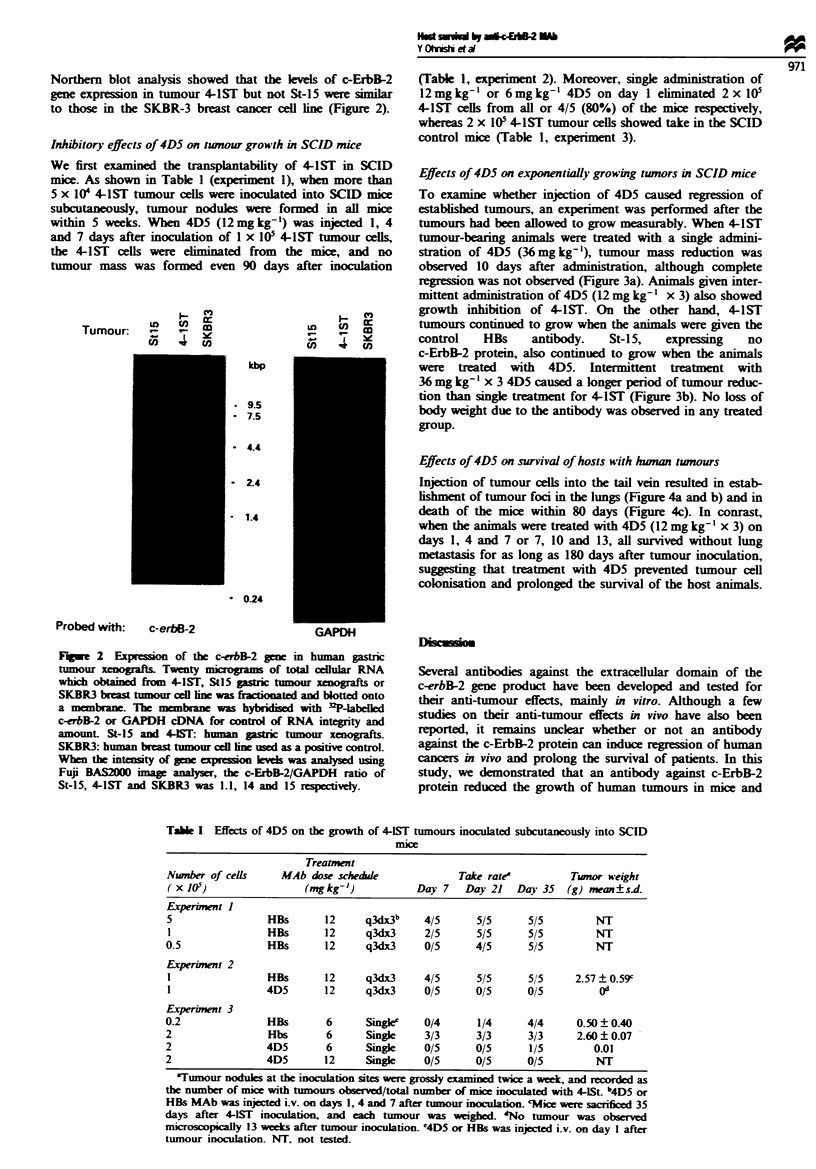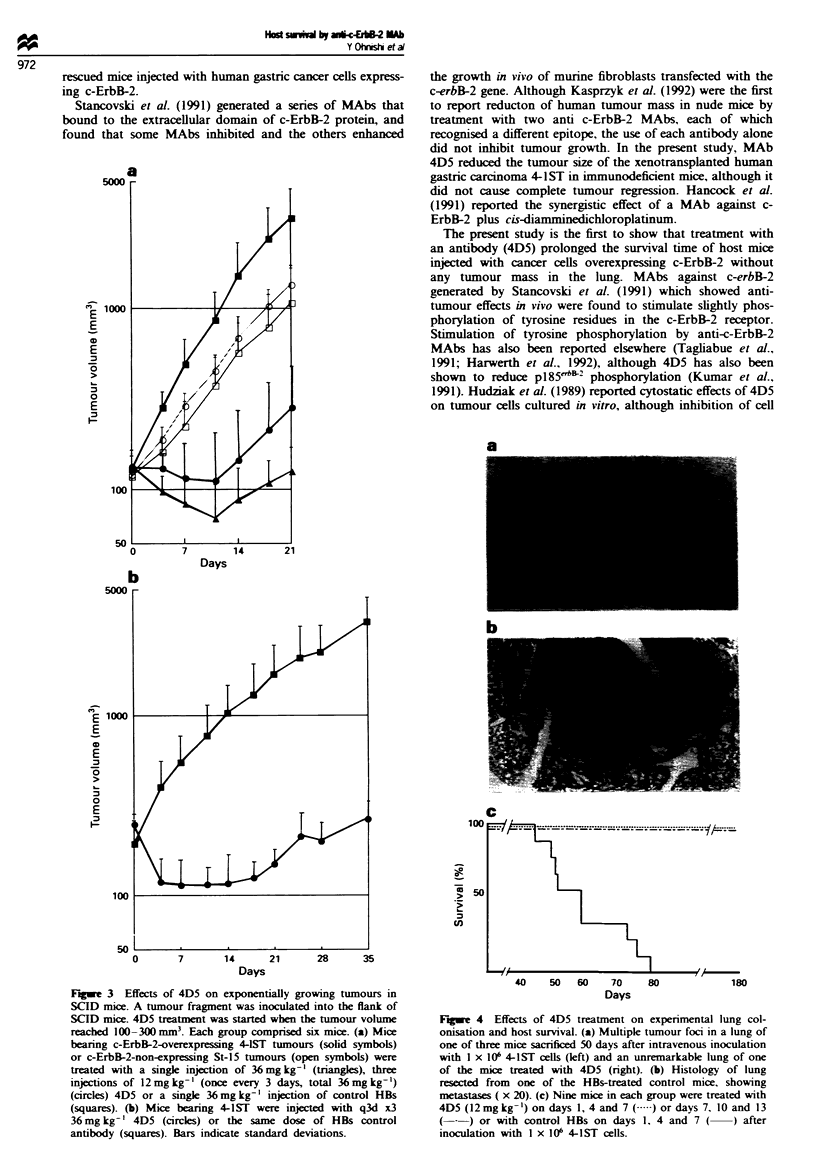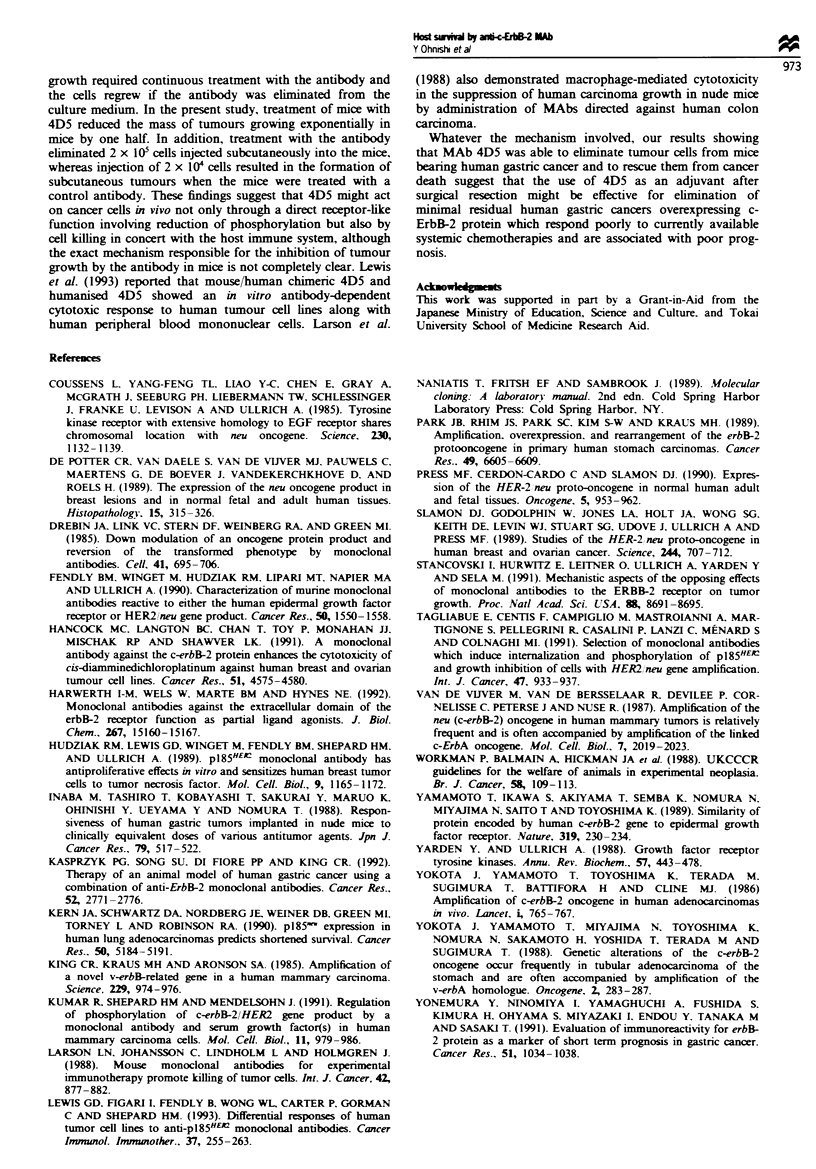# Prolonged survival of mice with human gastric cancer treated with an anti-c-ErbB-2 monoclonal antibody.

**DOI:** 10.1038/bjc.1995.187

**Published:** 1995-05

**Authors:** Y. Ohnishi, H. Nakamura, M. Yoshimura, Y. Tokuda, M. Iwasawa, Y. Ueyama, N. Tamaoki, K. Shimamura

**Affiliations:** Central Institute for Experimental Animals, Kawasaki, Japan.

## Abstract

**Images:**


British Journal of Cancer (1995) 71, 969-973

© 1995 Stockton Press All rights reserved 0007-0920/95 $12.00

### Prolonged survival of mice with human gastric cancer treated with an anti-c-ErbB-2 monoclonal antibody

Y Ohnishi[1], H Nakamura[2], M Yoshimura[1], Y Tokuda[3], M Iwasawa[3], Y Ueyama[1,4,5], N Tamaoki[4] and K Shimamura[4,5]

[1]*Central Institute for Experimental Animals, 1340 Nogawa, Miyamae-ku, Kawasaki 216, Japan;* [2]*Yokohama Research Center, Mitsubishi Chemical Corporation, 1000 Kamoshida-cho, Aoba-ku, Yokohama, 227, Japan;* [3]*Department of Surgery and* [4]*Pathology, Tokai University School of Medicine, Bohseidai, Isehara, 259-11, Japan;* [5]*Kanagawa Academy of Science and Technology, 3-2-1 Sakato, Takatsu-ku, Kawasaki 213, Japan.*

**Summary** A monoclonal antibody (MAb), 4D5, specifically recognising an extracellular epitope of the c-ErbB-2 protein, inhibited the growth of human gastric cancer overexpressing c-ErbB-2 severe combined immunodeficient (SCID) mice. This antibody also reduced the mass of established tumours xenografted into SCID mice, whereas gastric cancer not expressing c-ErbB-2 exhibited no regression in response to 4D5 treatment. In addition, administration of 4D5 prevented colonisation of cancer cells and prolonged the survival of host SCID mice inoculated i.v. with c-ErbB-2-overexpressing tumour cells. This is the first reported study to show that treatment with a single antibody specific to c-ErbB-2 prolongs the survival of host SCID mice bearing xenotransplanted tumours.

**Keywords:** c-ErbB-2; SCID mouse; human gastricarcinoma; monoclonal antibody; immunotherapy

The c-*erb*B-2/HER-2 proto-oncogene encodes a receptor-type tyrosine kinase (Yarden and Ullrich, 1988) related to, but different from, epidermal growth factor receptor (Coussens *et al.*, 1985; Yamamoto *et al.*, 1986). The c-ErbB-2 protein is expressed on the cell surface and consists of extracellular, transmembrane and intracellular domains, the last possessing kinase activity and autophosphorylation sites. Appreciable amplification and/or overexpression of this gene has been demonstrated in adenocarcinoma of the breast, ovary, lung and stomach (King *et al.*, 1985; Yokota *et al.*, 1986; van de Vijver *et al.*, 1987; Slamon *et al.*, 1989; Kern *et al.*, 1990). Amplification and/or overexpression was found in 8–40% of gastric carcinoma patients and was linked to low survival rates in the patients (Yokota *et al.*, 1988; Park *et al.*, 1989; Yomemura *et al.*, 1991). c-ErbB-2 is weakly expressed in normal tissues of adults (De Potter *et al.*, 1989; Press *et al.*, 1990). From the above findings, the c-ErbB-2 protein is thought to be a good target for antibody therapy of cancers showing overexpression. Several series of MAbs recognising the extracellular domain of the c-ErbB-2 protein have been tested for their anti-human tumour effects, mainly *in vitro*, and were found to produce inhibitory effects on cancer cell lines overexpressing c-ErbB-2 (Drebin *et al.*, 1985; Hudziak *et al.*, 1989; Hancock *et al.*, 1991; Stancovski *et al.*, 1991; Tagliabue *et al.*, 1991; Harwerth *et al.*, 1992; Kasprzyk *et al.*, 1992). Only a few reports of the *in vivo* effects of MAbs on tumours expressing the c-*erb*B-2 product have been published. Stancovski *et al.*, (1991) reported somewhat conflicting results on the *in vivo* effects of these MAbs on proliferation of murine fibroblasts transfected with the c-*erb*B-2 gene. Only Kasprzyk *et al.*,. (1992) have demonstrated regression of human tumours in nude mice by treatment with two different anti-c-ErbB-2 MAbs, although each antibody alone was not inhibitory. Whether or not the use of a single MAb is effective *in vivo* for inducing human tumour regression and improving host survival and the mechanisms involved in tumour regression *in vivo* remain unclear. A MAb against the c-ErbB-2 protein, 4D5, which was generated by Hudziak *et al.* (1989), has been reported to show cytostatic effects *in vitro* on several human breast cancer cells (Fendly *et al.*, 1990) as well as reduction of p185$^{erbB\text{-}2}$ phosphorylation (Kumar *et al.*, 1991). To study the *in vivo* effects of MAb 4D5 directed against the c-ErbB-2 protein on human tumour regression and host survival, the extent of tumour mass reduction and survival rate were determined in treated *vs* untreated mice bearing gastric cancer overexpressing c-ErbB-2.

### Materials and methods

### *Animals*

Balb/cA-*nu* mice were obtained from CLEA Japan *Inc* (Tokyo, Japan). C.B-17-*scid* mice were a gift from Dr GC Bosma (Fox Chase Cancer Center, Philadelphia, PA, USA) and bred in our animal quarters. The mice were used at 6–8 weeks of age in accordance with the animal care guidelines of the Central Institute for Experimental Animals (CIEA), including animal anaesthesia procedures. We performed periodic microbiological monitoring to confirm that the mice were kept under specific pathogen-free conditions during the experiment.

### *Cell lines and xenotransplanted tumour lines*

The human breast cancer cell line SK-BR-3 was obtained from the American Type Culture Collection (Rockville, MD, USA) and maintained in Dulbecco's modified Eagle medium (Sigma, St Louis, MO, USA) with 10% fetal bovine serum (FBS, Flow Laboratories, McLean, VA, USA). Human gastric carcinoma xenografts 4-1ST and St-15 were maintained at the CIEA by serial inoculation in Balb/cA nude mice.

### *Anti-c-ErbB-2 antibody and control antibody for* in vivo *anti-tumour assay*

A murine MAb, 4D5, recognising the extracellular domain of the c-ErbB-2 protein, was generated by Hudziak *et al.*, (1989) and supplied by Genentech. A class-matched MAb, HBs, recognising the surface antigen of hepatitis B virus, was used as the control.

### *Immunoblot analysis*

Tumour samples stored in liquid nitrogen were lysed in lysis buffer containing 20 mM Hepes pH 7.2, 1% Triton X-100,

Correspondence: K Shimamura
Received 2 June 1994; revised 12 December 1994; accepted 15 December 1994

10% glycerol, 1 mM EDTA, 1 mM sodium vanadate, 1 mM phenylmethylsulphonyl fluoride and 1 $\mu g\ ml^{-1}$ aprotinin in microtubes using a micropestle. The lysates were cleared by centrifugation, and 500 $\mu g$ of each lysate was immunoprecipitated with 10 $\mu$ of 4D5 or HBs control MAb. The protein was fractionated in 7.5% SDS- PAGE, transferred to an Immobilon membrane (Millipore Japan, Tokyo), probed with an anti-HER2/c-ErbB-2 antibody, CB11 (Novocastra Labs, UK) or an anti-phosphotyrosine antibody, 5E3 (Fendly *et al.*, 1990), and then treated with horseradish peroxidase (HRP)-conjugated anti-mouse IgG. Visualisation was performed using an ECL system (Amersham Japan, Tokyo).

*Northern blot analysis*

Northern blot analysis was performed using the method reported by Maniatis *et al.* (1989). Total cellular RNA (20 $\mu g$) was fractionated in a 1.0% agarose gel containing 6% formaldehyde and blotted onto a transfer membrane (DuPont-NEN, Boston, MA, USA). The membrane was hybridised with a $^{32}$p- labelled *XhoI*–*KpnI* fragment of *HER2*/c-*erb*B-2 cDNA (Coussens *et al.*, 1985), then rehybridised with a chicken glyceraldehyde phosphate (GAPDH) cDNA probe, and was analyzed using Fuji BAS2000 image analyser (Fuji Film, Tokyo, Japan).

*Preparation of single-cell suspensions*

Solid tumours in nude mice were resected, finely dispersed using scissors, incubated in Hanks' balanced salt solution containing 0.05% pronase (Boehringer Mannheim, Germany), 0.02% collagenase type 1 (Sigma, St Louis, MO, USA) and 0.02% DNAse I (Sigma) at 37°C for 30 min, and passed finally through a nylon mesh to prepare single-cell suspensions.

*Inhibition of tumour take*

Tumour cells were subcutaneously inoculated into the right flank of severe combined immunodeficient (SCID) mice, followed by i.v. injection of either 4D5 or HBs. Tumour mass formation at the inoculation site was observed twice a week, and tumour weight was measured on the day that the mice were sacrificed.

*Exponential growth model*

Effects on the tumours in the exponential growth phase were examined by the method described previously (Inaba *et al.*, 1988) using SCID mice instead of nude mice. In brief, a tumour fragment was inoculated subcutaneously into the right flank of SCID mice. Tumour size was measured twice a week with calipers, and tumour volume was calculated according to the formula tumour volume (mm$^3$) = length $\times$ (width)$^2$/2. The mice were randomly divided into experimental groups when each tumour had reached a palpable size (100–300 mm$^3$), and 4D5 or HBs MAb was then injected intravenously into mice in each group. Statistical significance of differences was determined by the Mann–Whitney *U*-test.

*Experimental lung metastasis model*

One million 4-1ST cells were inoculated i.v. into the tail veins of SCID mice, followed by i.v. injection of MAbs on days 1, 4 and 7 or 7, 10 and 13. Survival periods of mice were observed for 180 days. When the animals showed severe wasting and were apparently moribund owing to tumour growth in the lung, the animals were not observed further and the day of sacrifice was recorded according to the UKCCCR guidelines (Workman *et al.*, 1988). Surviving animals were sacrificed at the end of the experiment and sectioned for microscopic examination of tumour foci. In a preliminary experiment, mice inokculated with $1 \times 10^6$ 4-1ST cells were sacrificed on day 50 to confirm the presence of tumour foci in the lung.

### Results

*Expression of c-ErbB-2 product in human gastric carcinoma xenografts, 4-1ST and St-15*

To select a suitable human tumour line as an *in vivo* model for therapy using anti-c-ErbB-2 MAb, we first screened the immunoreactivity with an anti-c-ErbB-2 polyclonal antibody of 18 human gastric carcinomas xenotransplanted into nude mice by immunoblotting (data not shown). Among these tumours, the 4-1ST tumour line derived from a poorly differentiated adenocarcinoma revealed a clear band at a molecular mass of 185 kDa, the reported molecular mass of p185$^{erbB-2}$, whereas St-15 showed no reactive product (Figure 1). 4-1ST also showed many bands with a lower molecular mass than 185 kDa. We assume that these lower bands were caused by reaction with the degraded c-ErbB-2 product, since the xenografted tumours contained necrotic tissue in their centre. An anti-phosphotyrosine antibody, 5E3, reacted with a 185 kDa protein immunoprecipitated from the 4-1ST tumour lysate with 4D5, suggesting that p185$^{erbB-2}$ overexpressed in this tumour is phosphorylated (Figure 1).

**Figure 1** Immunoblot analysis of c-ErbB-2 protein in human gastric tumour xenografts. Lysates from 4-1ST and St-15 human gastric tumours were immunoprecipitated with 4D5 or the control MAb HBs. The immunoprecipitated proteins were fractionated by SDS–PAGE, and transferred to a membrane. The membrane was probed with anti-HER2/ErbB-2 antibody, CB11 (left), or anti-phosphotyrosine antibody, 5E3 (right), and then with HRP-conjugated anti-mouse antibody. Reactions were visualised using the ECL system (Amersham Japan). Arrowheads indicated p185$^{c-erbB-2}$ and tyrosine-phosphorylated p185$^{c-erbB-2}$. The small arrow shows the Ig heavy chain used in immunoprecipitation.

Northern blot analysis showed that the levels of c-ErbB-2 gene expression in tumour 4-1ST but not St-15 were similar to those in the SKBR-3 breast cancer cell line (Figure 2).

*Inhibitory effects of 4D5 on tumour growth in SCID mice*

We first examined the transplantability of 4-1ST in SCID mice. As shown in Table 1 (experiment 1), when more than $5 \times 10^4$ 4-1ST tumour cells were inoculated into SCID mice subcutaneously, tumour nodules were formed in all mice within 5 weeks. When 4D5 (12 mg $kg^{-1}$) was injected 1, 4 and 7 days after inoculation of $1 \times 10^5$ 4-1ST tumour cells, the 4-1ST cells were eliminated from the mice, and no tumour mass was formed even 90 days after inoculation

**Figure 2** Expression of the c-*erb*B-2 gene in human gastric tumour xenografts. Twenty micrograms of total cellular RNA which obtained from 4-1ST, St15 gastric tumour xenografts or SKBR3 breast tumour cell line was fractionated and blotted onto a membrane. The membrane was hybridised with $^{32}P$-labelled c-*erb*B-2 or GAPDH cDNA for control of RNA integrity and amount. St-15 and 4-1ST: human gastric tumour xenografts. SKBR3: human breast tumour cell line used as a positive control. When the intensity of gene expression levels was analysed using Fuji BAS2000 image analyser, the c-ErbB-2/GAPDH ratio of St-15, 4-1ST and SKBR3 was 1.1, 14 and 15 respectively.

(Table 1, experiment 2). Moreover, single administration of 12 mg $kg^{-1}$ or 6 mg $kg^{-1}$ 4D5 on day 1 eliminated $2 \times 10^5$ 4-1ST cells from all or 4/5 (80%) of the mice respectively, whereas $2 \times 10^5$ 4-1ST tumour cells showed take in the SCID control mice (Table 1, experiment 3).

*Effects of 4D5 on exponentially growing tumors in SCID mice*

To examine whether injection of 4D5 caused regression of established tumours, an experiment was performed after the tumours had been allowed to grow measurably. When 4-1ST tumour-bearing animals were treated with a single administration of 4D5 (36 mg $kg^{-1}$), tumour mass reduction was observed 10 days after administration, although complete regression was not observed (Figure 3a). Animals given intermittent administration of 4D5 (12 mg $kg^{-1}$ × 3) also showed growth inhibition of 4-1ST. On the other hand, 4-1ST tumours continued to grow when the animals were given the control HBs antibody. St-15, expressing no c-ErbB-2 protein, also continued to grow when the animals were treated with 4D5. Intermittent treatment with 36 mg $kg^{-1}$ × 3 4D5 caused a longer period of tumour reduction than single treatment for 4-1ST (Figure 3b). No loss of body weight due to the antibody was observed in any treated group.

*Effects of 4D5 on survival of hosts with human tumours*

Injection of tumour cells into the tail vein resulted in establishment of tumour foci in the lungs (Figure 4a and b) and in death of the mice within 80 days (Figure 4c). In conrast, when the animals were treated with 4D5 (12 mg $kg^{-1}$ × 3) on days 1, 4 and 7 or 7, 10 and 13, all survived without lung metastasis for as long as 180 days after tumour inoculation, suggesting that treatment with 4D5 prevented tumour cell colonisation and prolonged the survival of the host animals.

### Discussion

Several antibodies against the extracellular domain of the c-*erb*B-2 gene product have been developed and tested for their anti-tumour effects, mainly *in vitro*. Although a few studies on their anti-tumour effects *in vivo* have also been reported, it remains unclear whether or not an antibody against the c-ErbB-2 protein can induce regression of human cancers *in vivo* and prolong the survival of patients. In this study, we demonstrated that an antibody against c-ErbB-2 protein reduced the growth of human tumours in mice and

**Table I** Effects of 4D5 on the growth of 4-1ST tumours inoculated subcutaneously into SCID mice

| *Number of cells* (× $10^5$) | *Treatment MAb dose schedule* (mg $kg^{-1}$) | | | *Take rate*[a] Day 7 | Day 21 | Day 35 | *Tumor weight (g) mean±s.d.* |
|---|---|---|---|---|---|---|---|
| *Experiment 1* | | | | | | | |
| 5 | HBs | 12 | q3dx3[b] | 4/5 | 5/5 | 5/5 | NT |
| 1 | HBs | 12 | q3dx3 | 2/5 | 5/5 | 5/5 | NT |
| 0.5 | HBs | 12 | q3dx3 | 0/5 | 4/5 | 5/5 | NT |
| *Experiment 2* | | | | | | | |
| 1 | HBs | 12 | q3dx3 | 4/5 | 5/5 | 5/5 | 2.57 ± 0.59[c] |
| 1 | 4D5 | 12 | q3dx3 | 0/5 | 0/5 | 0/5 | 0[d] |
| *Experiment 3* | | | | | | | |
| 0.2 | HBs | 6 | Single[e] | 0/4 | 1/4 | 4/4 | 0.50 ± 0.40 |
| 2 | Hbs | 6 | Single | 3/3 | 3/3 | 3/3 | 2.60 ± 0.07 |
| 2 | 4D5 | 6 | Single | 0/5 | 0/5 | 1/5 | 0.01 |
| 2 | 4D5 | 12 | Single | 0/5 | 0/5 | 0/5 | NT |

[a]Tumour nodules at the inoculation sites were grossly examined twice a week, and recorded as the number of mice with tumours observed/total number of mice inoculated with 4-1St. [b]4D5 or HBs MAb was injected i.v. on days 1, 4 and 7 after tumour inoculation. [c]Mice were sacrificed 35 days after 4-1ST inoculation, and each tumour was weighed. [d]No tumour was observed microscopically 13 weeks after tumour inoculation. [e]4D5 or HBs was injected i.v. on day 1 after tumour inoculation. NT, not tested.

rescued mice injected with human gastric cancer cells expressing c-ErbB-2.

Stancovski *et al.* (1991) generated a series of MAbs that bound to the extracellular domain of c-ErbB-2 protein, and found that some MAbs inhibited and the others enhanced the growth *in vivo* of murine fibroblasts transfected with the c-*erb*B-2 gene. Although Kasprzyk *et al.* (1992) were the first to report reducton of human tumour mass in nude mice by treatment with two anti c-ErbB-2 MAbs, each of which recognised a different epitope, the use of each antibody alone did not inhibit tumour growth. In the present study, MAb 4D5 reduced the tumour size of the xenotransplanted human gastric carcinoma 4-1ST in immunodeficient mice, although it did not cause complete tumour regression. Hancock *et al.* (1991) reported the synergistic effect of a MAb against c-ErbB-2 plus *cis*-diamminedichloroplatinum.

The present study is the first to show that treatment with an antibody (4D5) prolonged the survival time of host mice injected with cancer cells overexpressing c-ErbB-2 without any tumour mass in the lung. MAbs against c-*erb*B-2 generated by Stancovski *et al.* (1991) which showed anti-tumour effects *in vivo* were found to stimulate slightly phosphorylation of tyrosine residues in the c-ErbB-2 receptor. Stimulation of tyrosine phosphorylation by anti-c-ErbB-2 MAbs has also been reported elsewhere (Tagliabue *et al.*, 1991; Harwerth *et al.*, 1992), although 4D5 has also been shown to reduce $p185^{erbB-2}$ phosphorylation (Kumar *et al.*, 1991). Hudziak *et al.* (1989) reported cytostatic effects of 4D5 on tumour cells cultured *in vitro*, although inhibition of cell

**Figure 3** Effects of 4D5 on exponentially growing tumours in SCID mice. A tumour fragment was inoculated into the flank of SCID mice. 4D5 treatment was started when the tumour volume reached 100–300 mm³. Each group comprised six mice. (**a**) Mice bearing c-ErbB-2-overexpressing 4-1ST tumours (solid symbols) or c-ErbB-2-non-expressing St-15 tumours (open symbols) were treated with a single injection of 36 mg $kg^{-1}$ (triangles), three injections of 12 mg $kg^{-1}$ (once every 3 days, total 36 mg $kg^{-1}$) (circles) 4D5 or a single 36 mg $kg^{-1}$ injection of control HBs (squares). (**b**) Mice bearing 4-1ST were injected with q3d x3 36 mg $kg^{-1}$ 4D5 (circles) or the same dose of HBs control antibody (squares). Bars indicate standard deviations.

**Figure 4** Effects of 4D5 treatment on experimental lung colonisation and host survival. (**a**) Multiple tumour foci in a lung of one of three mice sacrificed 50 days after intravenous inoculation with 1 × $10^6$ 4-1ST cells (left) and an unremarkable lung of one of the mice treated with 4D5 (right). (**b**) Histology of lung resected from one of the HBs-treated control mice, showing metastases ( × 20). (**c**) Nine mice in each group were treated with 4D5 (12 mg $kg^{-1}$) on days 1, 4 and 7 (·····) or days 7, 10 and 13 (—·—) or with control HBs on days 1, 4 and 7 (——) after inoculation with 1 × $10^6$ 4-1ST cells.

growth required continuous treatment with the antibody and the cells regrew if the antibody was eliminated from the culture medium. In the present study, treatment of mice with 4D5 reduced the mass of tumours growing exponentially in mice by one half. In addition, treatment with the antibody eliminated $2 \times 10^5$ cells injected subcutaneously into the mice, whereas injection of $2 \times 10^4$ cells resulted in the formation of subcutaneous tumours when the mice were treated with a control antibody. These findings suggest that 4D5 might act on cancer cells *in vivo* not only through a direct receptor-like function involving reduction of phosphorylation but also by cell killing in concert with the host immune system, although the exact mechanism responsible for the inhibition of tumour growth by the antibody in mice is not completely clear. Lewis *et al.* (1993) reported that mouse/human chimeric 4D5 and humanised 4D5 showed an *in vitro* antibody-dependent cytotoxic response to human tumour cell lines along with human peripheral blood mononuclear cells. Larson *et al.* (1988) also demonstrated macrophage-mediated cytotoxicity in the suppression of human carcinoma growth in nude mice by administration of MAbs directed against human colon carcinoma.

Whatever the mechanism involved, our results showing that MAb 4D5 was able to eliminate tumour cells from mice bearing human gastric cancer and to rescue them from cancer death suggest that the use of 4D5 as an adjuvant after surgical resection might be effective for elimination of minimal residual human gastric cancers overexpressing c-ErbB-2 protein which respond poorly to currently available systemic chemotherapies and are associated with poor prognosis.

**Acknowledgments**
This work was supported in part by a Grant-in-Aid from the Japanese Ministry of Education, Science and Culture, and Tokai University School of Medicine Research Aid.

**References**

COUSSENS L, YANG-FENG TL, LIAO Y-C, CHEN E, GRAY A, McGRATH J, SEEBURG PH, LIEBERMANN TW, SCHLESSINGER J, FRANKE U, LEVISON A AND ULLRICH A. (1985). Tyrosine kinase receptor with extensive homology to EGF receptor shares chromosomal location with *neu* oncogene. *Science*, **230**, 1132–1139.

DE POTTER CR, VAN DAELE S, VAN DE VIJVER MJ, PAUWELS C, MAERTENS G, DE BOEVER J, VANDEKERCHKHOVE D, AND ROELS H. (1989). The expression of the *neu* oncogene product in breast lesions and in normal fetal and adult human tissues. *Histopathology*, **15**, 315–326.

DREBIN JA, LINK VC, STERN DF, WEINBERG RA, AND GREEN MI. (1985). Down modulation of an oncogene protein product and reversion of the transformed phenotype by monoclonal antibodies. *Cell*, **41**, 695–706.

FENDLY BM, WINGET M, HUDZIAK RM, LIPARI MT, NAPIER MA AND ULLRICH A. (1990). Characterization of murine monoclonal antibodies reactive to either the human epidermal growth factor receptor or HER2/neu gene product. *Cancer Res.*, **50**, 1550–1558.

HANCOCK MC, LANGTON BC, CHAN T, TOY P, MONAHAN JJ, MISCHAK RP AND SHAWVER LK. (1991). A monoclonal antibody against the c-*erb*B-2 protein enhances the cytotoxicity of *cis*-diamminedichloroplatinum against human breast and ovarian tumour cell lines. *Cancer Res.*, **51**, 4575–4580.

HARWERTH I-M, WELS W, MARTE BM AND HYNES NE. (1992). Monoclonal antibodies against the extracellular domain of the erbB-2 receptor function as partial ligand agonists. *J. Biol. Chem.*, **267**, 15160–15167.

HUDZIAK RM, LEWIS GD, WINGET M, FENDLY BM, SHEPARD HM, AND ULLRICH A. (1989). p185$^{HER2}$ monoclonal antibody has antiproliferative effects *in vitro* and sensitizes human breast tumor cells to tumor necrosis factor. *Mol. Cell. Biol.*, **9**, 1165–1172.

INABA M, TASHIRO T, KOBAYASHI T, SAKURAI Y, MARUO K, OHINISHI Y, UEYAMA Y AND NOMURA T. (1988). Responsiveness of human gastric tumors implanted in nude mice to clinically equivalent doses of various antitumor agents. *Jpn J. Cancer Res.*, **79**, 517–522.

KASPRZYK PG, SONG SU, DI FIORE PP AND KING CR. (1992). Therapy of an animal model of human gastric cancer using a combination of anti-*Erb*B-2 monoclonal antibodies. *Cancer Res.*, **52**, 2771–2776.

KERN JA, SCHWARTZ DA, NORDBERG JE, WEINER DB, GREEN MI, TORNEY L AND ROBINSON RA. (1990). p185$^{neu}$ expression in human lung adenocarcinomas predicts shortened survival. *Cancer Res.*, **50**, 5184–5191.

KING CR, KRAUS MH AND ARONSON SA. (1985). Amplification of a novel v-*erb*B-related gene in a human mammary carcinoma. *Science*, **229**, 974–976.

KUMAR R, SHEPARD HM AND MENDELSOHN J. (1991). Regulation of phosphorylation of c-*erb*B-2/*HER2* gene product by a monoclonal antibody and serum growth factor(s) in human mammary carcinoma cells. *Mol. Cell. Biol.*, **11**, 979–986.

LARSON LN, JOHANSSON C, LINDHOLM L AND HOLMGREN J. (1988). Mouse monoclonal antibodies for experimental immunotherapy promote killing of tumor cells. *Int. J. Cancer*, **42**, 877–882.

LEWIS GD, FIGARI I, FENDLY B, WONG WL, CARTER P, GORMAN C AND SHEPARD HM. (1993). Differential responses of human tumor cell lines to anti-p185$^{HER2}$ monoclonal antibodies. *Cancer Immunol. Immunother.*, **37**, 255–263.

NANIATIS T, FRITSH EF AND SAMBROOK J. (1989). *Molecular cloning: A laboratory manual*. 2nd edn. Cold Spring Harbor Laboratory Press: Cold Spring Harbor, NY.

PARK JB, RHIM JS, PARK SC, KIM S-W AND KRAUS MH. (1989). Amplification, overexpression, and rearrangement of the *erb*B-2 protooncogene in primary human stomach carcinomas. *Cancer Res.*, **49**, 6605–6609.

PRESS MF, CERDON-CARDO C AND SLAMON DJ. (1990). Expression of the *HER-2/neu* proto-oncogene in normal human adult and fetal tissues. *Oncogene*, **5**, 953–962.

SLAMON DJ, GODOLPHIN W, JONES LA, HOLT JA, WONG SG, KEITH DE, LEVIN WJ, STUART SG, UDOVE J, ULLRICH A AND PRESS MF. (1989). Studies of the *HER-2/neu* proto-oncogene in human breast and ovarian cancer. *Science*, **244**, 707–712.

STANCOVSKI I, HURWITZ E, LEITNER O, ULLRICH A, YARDEN Y AND SELA M. (1991). Mechanistic aspects of the opposing effects of monoclonal antibodies to the ERBB-2 receptor on tumor growth. *Proc. Natl Acad. Sci. USA*, **88**, 8691–8695.

TAGLIABUE E, CENTIS F, CAMPIGLIO M, MASTROIANNI A, MARTIGNONE S, PELLEGRINI R, CASALINI P, LANZI C, MÉNARD S AND COLNAGHI MI. (1991). Selection of monoclonal antibodies which induce internalization and phosphorylation of p185$^{HER2}$ and growth inhibition of cells with *HER2/neu* gene amplification. *Int. J. Cancer*, **47**, 933–937.

VAN DE VIJVER M, VAN DE BERSSELAAR R, DEVILEE P, CORNELISSE C, PETERSE J AND NUSE R. (1987). Amplification of the *neu* (c-*erb*B-2) oncogene in human mammary tumors is relatively frequent and is often accompanied by amplification of the linked c-*Erb*A oncogene. *Mol. Cell. Biol.*, **7**, 2019–2023.

WORKMAN P, BALMAIN A, HICKMAN JA *et al.* (1988). UKCCCR guidelines for the welfare of animals in experimental neoplasia. *Br. J. Cancer*, **58**, 109–113.

YAMAMOTO T, IKAWA S, AKIYAMA T, SEMBA K, NOMURA N, MIYAJIMA N, SAITO T AND TOYOSHIMA K. (1989). Similarity of protein encoded by human c-*erb*B-2 gene to epidermal growth factor receptor. *Nature*, **319**, 230–234.

YARDEN Y. AND ULLRICH A. (1988). Growth factor receptor tyrosine kinases. *Annu. Rev. Biochem.*, **57**, 443–478.

YOKOTA J, YAMAMOTO T, TOYOSHIMA K, TERADA M, SUGIMURA T, BATTIFORA H AND CLINE MJ. (1986) Amplification of c-*erb*B-2 oncogene in human adenocarcinomas *in vivo*. *Lancet*, **i**, 765–767.

YOKOTA J, YAMAMOTO T, MIYAJIMA N, TOYOSHIMA K, NOMURA N, SAKAMOTO H, YOSHIDA T, TERADA M AND SUGIMURA T. (1988). Genetic alterations of the c-*erb*B-2 oncogene occur frequently in tubular adenocarcinoma of the stomach and are often accompanied by amplification of the v-*erb*A homologue. *Oncogene*, **2**, 283–287.

YONEMURA Y, NINOMIYA I, YAMAGHUCHI A, FUSHIDA S, KIMURA H, OHYAMA S, MIYAZAKI I, ENDOU Y, TANAKA M AND SASAKI T. (1991). Evaluation of immunoreactivity for *erb*B-2 protein as a marker of short term prognosis in gastric cancer. *Cancer Res.*, **51**, 1034–1038.